# Allometric Scaling of Mutual Information in Complex Networks: A Conceptual Framework and Empirical Approach

**DOI:** 10.3390/e22020206

**Published:** 2020-02-12

**Authors:** Eduardo Viegas, Hayato Goto, Yuh Kobayashi, Misako Takayasu, Hideki Takayasu, Henrik Jeldtoft Jensen

**Affiliations:** 1 Centre for Complexity Science and Department of Mathematics, Imperial College London, London SW7 2AZ, UK; h.goto@imperial.ac.uk (H.G.); h.jensen@imperial.ac.uk (H.J.J.); 2 Institute of Innovative Research, Tokyo Institute of Technology, 4259, Nagatsuta-cho, Yokohama 226-8502, Japan; kobayashi.y.bz@m.titech.ac.jp (Y.K.); takayasu.m.aa@m.titech.ac.jp (M.T.); takayasu.h.aa@m.titech.ac.jp (H.T.); 3 Department of Mathematical and Computing Science, School of Computing, Tokyo Institute of Technology, Yokohama 226-8502, Japan; 4 Sony Computer Science Laboratories, 3-14-13, Higashi-Gotanda, Shinagawa-ku, Tokyo 141-0022, Japan

**Keywords:** complexity science, information theory, economic complexity, evolutionary dynamics, network theory

## Abstract

Complexity and information theory are two very valuable but distinct fields of research, yet sharing the same roots. Here, we develop a complexity framework inspired by the allometric scaling laws of living biological systems in order to evaluate the structural features of networks. This is done by aligning the fundamental building blocks of information theory (entropy and mutual information) with the core concepts in network science such as the preferential attachment and degree correlations. In doing so, we are able to articulate the meaning and significance of mutual information as a comparative analysis tool for network activity. When adapting and applying the framework to the specific context of the business ecosystem of Japanese firms, we are able to highlight the key structural differences and efficiency levels of the economic activities within each prefecture in Japan. Moreover, we propose a method to quantify the distance of an economic system to its efficient free market configuration by distinguishing and quantifying two particular types of mutual information, total and structural.

## 1. Introduction

One can argue that statistical physics and theoretical computing are the common roots feeding the science branches of complexity and information theory, as attested by the early exchanges of ideas between von Neumann and Shannon. Whereas the latter was more preoccupied in quantitatively measuring the encoding and transmission of information [1], the former (as articulated by the automata theory) had its focus on information replication with mutation but without generating tendencies [2] (i.e., self replication, or ‘evolution’) as well as the processing functions at an individual and aggregated level [3] (i.e., general automata and basic organs, or ‘emerging scaling properties of a network structure’). Since then, these aforementioned fields of science have progressed significantly, and developed to an extent that they seem to bear little in common. Yet, significant insight can be obtained if one were to recombine these fields and develop a framework articulating the link between the emerging structural properties of a network and the flow, or encoding, of information within [4,5]. Given the importance of evolution and scaling to such framework, useful mathematical methods can be applied by borrowing concepts from the biological, natural world, in particular the diversity of species and allometric scaling [6,7,8].

In precis, this is the core motivation and aim of our research. Here, we create a method within the context of a network flow of resources to measure two fundamental quantities underpinning information theory, namely entropy and mutual information. This method is then wrapped into a framework that draws parallels to the biological context of body growth and allometric scaling so that the meaning and significance of mutual information within this construction can be better understood and intuitively rationalised.

### 1.1. The Context

From an empirical perspective, we make use of the real ‘Interfirm Business Transaction Network’ within Japan, consisting of detailed granular level transaction data among over 600,000 firms during a 25-year period, 1994 to 2018, provided by Teikoku Databank. This rich dataset allows the breakdown of an extensive network into smaller subgraphs, at a prefecture level, so that a dynamic comparison between different segments of the network can be carried out. Previous works on the real trade Japanese network [9,10,11] are centred on the system dynamics surrounding the formation of the network, as well as the structural analysis [12] including studies on allometric scaling [13] of quantities such as sales and income. Our research, however, adopted a fundamentally distinct approach as it seeks to answer different questions. Here, we are less preoccupied with the dynamics of network formation. Instead, our focus is mostly on constructing a framework based on mutual information and resource usage efficiency, akin to metabolism, that allows for a direct comparison of different regional economic activities, in this case of 47 Japanese prefectures. We emphasise that we have in mind that mutual information represents effective cash flow (i.e., the movement of resources) between companies, since the scaling properties of the real trade network, referred above, allow us to make use of the degree of a company as a proxy that can be measured directly from the data, so that we avoid issues surrounding cash flow estimation.

Furthermore, we note that our work bears some similarity to existing ecological network analysis for economic systems, where resources defined as currency cash flows are used as the basis for calculating entropy and mutual information [14]. However, data, methods, and objectives fundamentally differ. Importantly, we are not preoccupied with measuring economic development. Instead, our focus is on the understanding of structural evolution of trade networks as described above.

Since our work is applied to a real countrywide economic system and financial network, it is a requirement for the conceptual framework to be adapted in order to incorporate key fundamental economic principles. This is to ensure that the concept of mutual information is aligned not only to biological metabolic rates but to the specifics and concrete elements of the network studied as well. Essentially, this means that the analogy to metabolic rate is further extended to define the average resources used by a company to generate new trades (and the related cashflows and income). In very simplified terms, since the focus here is not on detailed finance and economics, the metabolic rate can be generically equated to a cost to acquire new trades [15].

### 1.2. The Complexity Framework: Allometric Nature of Mutual Information

From a conceptual perspective, our framework can be regarded as a triangulation between concepts arising from three different fields of study: Network science, information theory, and economics.

Firstly, from a network science perspective, previous academic works show that the distribution of nodes and edges for the Japanese inter-firm trade network follows a power law distribution governed by mechanisms associated with a cumulative advantage [16] and preferential attachment [17]. Essentially, these mechanisms tend to lead, but not inevitably, to the formation of a disassortative network, essentially meaning that the average number of nodes connected to a specific selected node tends to decrease as the degree of the latter gets larger. Regardless of the specific mechanisms of a network, the power law structure will always tend to lead to a level of disassortativeness [18].

From an information theory perspective, it follows that an amount of mutual information will always be different from zero if the network is disassortative, simply as a result of the functional forms of entropy and mutual information. Therefore, within our framework, it is possible to break the computation of the mutual information into two separate but related components: The structural mutual information, SMI, and the total mutual information, *I*. The former solely relates to the degree distribution of the nodes within a given network, whereas the latter encompasses both the node degree distribution as well as the disassortativeness of the network.

Such distinction also fits well within the economics and finance perspective since SMI can be related to a theoretical ’free-market’, stock market-type configuration, whereas *I* is not only naturally associated with, but reflects, the real world situation.

Once the above is addressed, we overlay the biological dimension of our framework.

#### 1.2.1. Structural Mutual Information: SMI

The structural mutual information SMI is intended to capture the basic quantities held by the network simply as a result of the power law like degree distribution of companies, and their related sizes, within a given network. Essentially, we make use of the term ‘structural’ to refer to the basic existence of the nodes without taking into account the dynamics of the preferential attachment and cumulative advantage mechanism. The method is inspired by the allometric scaling and power laws in ecological systems. In particular, we make use here of the allometric scaling equation leading to an analogy whereby SMI and *I* for each prefecture can be related to the metabolic rates *B* of an individual which is known to scale with body size *M* as:

(1) $$B=Q_{1}RM^{b}$$

where the exponent b=3/4 has been suggested to describe a range of biological cases [6,8]. The two other elements, $Q_{1}R$ capture, essentially, the variability in resource supply rates as well as variables affecting body size and density.

#### 1.2.2. Total Mutual Information, I

Further extending our analogy, SMI is akin to the resting, or basal, metabolic rate. In contrast, the total mutual information *I* contains the additional thermal food and physical effects. Within our framework, these two additional effects represent activities comparable to the way that companies express trading preferences among themselves (i.e., the dynamics of preferential attachment and cumulative advantage). Therefore, these dynamics act as a multiplier to the core, structural mutual information.

### 1.3. The Economic Dimension

Given that companies always aim to increase profits by maximising income and minimising costs, it is only natural to reason that the dynamics of preferential attachment and cumulative advantage become a natural feature of general business dynamics [19]. Specifically, small companies with very limited resources would tend to be most efficient when selling all their output to a single (or at least very few) company in order to reduce costs. In contrast, larger companies with additional resources will be driven by income expansion and therefore are willing to trade across as many agents as possible.

Here, without making any judgement about merits and disadvantages of distinct economic systems, we note that a centralised style communist system can be regarded as an extreme case of preferential attachment since virtually almost all market agents will almost solely trade with the largest entities (i.e., governments and large public companies). Yet, the current Western ‘capitalist’ system also tends to lead to a virtual monopoly by the largest companies [9,20]. Therefore, one can reasonably argue that Western-style developed economies are no longer structurally capitalist, typically underpinned by a free market configuration. It is important to recognise, however, the fact that there is no single, or commonly accepted, definition of a ‘free market economy’ [21,22] within the field of economics. Therefore, an element of constrained licentia poetica will inevitably be required when attempting to define a theoretical free efficient market within the economic context. To mitigate the effects of such an issue, we specifically, and narrowly, interpret ‘free market economies’ to be those that structurally resemble the dynamics of organised markets, such as the stock markets. Within these markets, each unit traded, such as a single quantity stock or the minimum denomination of a bond is not dependent on any trait of the buyer or sell, or any other trade activity. Therefore, the preferential attachment mechanism is virtually absent, since the identity of the buyer is unlikely to be known by the seller and vice versa. Yet, a higher probability that a small agent will trade with larger agents, such as pension and investment funds, are still likely to exist simply due to sheer size. It is a fact that a large entity will have a higher number of trades, but will also be subject to some scaling of costs due to a higher activity.

Adapting the analogy for metabolic rates to the economic dimension, the structural mutual information SMI captures the mutual information solely arising as a result of the size of companies as if these companies were theoretically trading on a configuration similar, or akin, to stock markets, or ’free markets’. In order to measure this component, we randomised the real network, preserving the structure of the nodes (i.e., to total degree distributions) while minimising the effects of preferential attachment (i.e., degree correlations). In contrast, the mutual information *I* is directly derived from the real network configuration, which includes both the effect captured by the structural mutual information as well as the additional quantities arising from the dynamics of preferential attachment and cumulative advantage. By making this distinction, we are able to compare the efficiency of different Japanese prefectures and better understand the structure and activities of these prefectures.

## 2. Results

Our results are presented in three sections. Firstly, under the Data Analysis section, we present an analysis of the evolution of the entropies and mutual information for the Japanese prefectures over a 25-year period. This analysis is then enhanced and further analysed by layering the geographical dimension of the prefectures across Japan. The second section covers the results of applying the framework based on the allometric scaling of mutual information and related analogies to metabolism and biological systems to real world data. Thirdly, we zoom into a more microscopic level, specifically local interaction, analysing the formation and contribution to mutual information at a pointwise level, where the effects of the preferential attachment and cumulative advantage mechanisms can be clearly observed.

### 2.1. Data Analysis

By applying the grouping and coarse graining process as described in the Methods section, the values for entropy *H*, joint entropy *J*, and mutual information *I* for each prefecture were directly measured from real Japanese interfirm trade network data by making use of Equations (5), (6), and (7), respectively.

#### 2.1.1. Macro Features of Entropy and Mutual Information

**Figure 1:**
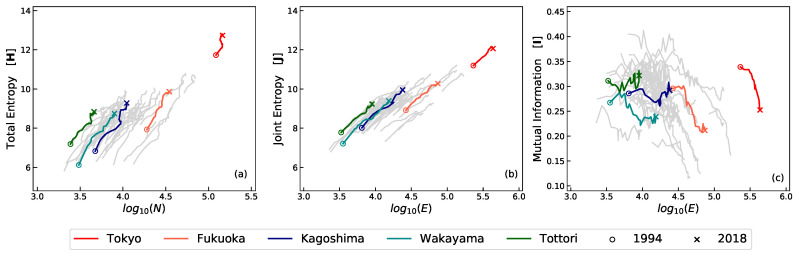
Entropy and mutual information prefectures in Japan between 1994 and 2018. Plot (**a**) shows the evolution of the total entropy *H* as a function of the total number of companies (nodes) |C| during the period 1994–2018. Similarly, plots (**b**,**c**) show the equivalent joint entropy *J* and mutual information *I* as a function of total number of edges *E*. Each grey line represents the path taken single prefecture in Japan during the period 1994–2018. The coloured lines highlighting representative prefectures, selected from the largest (i.e., Tokyo) to smallest (i.e., Tottori) in terms of GDP (gross domestic product) and maintaining similar ranking intervals in between (Fukuoka, Kagoshima, and Wakayama). The circles point to the year 1994 whereas the x-cross relates to 2018.

Consistently with established literature [18], the entropy, and the joint entropy, of the Japanese trade network in its totality or within each subgraph, i.e., the prefecture level, will tend to be higher as the system grows in size (i.e., increasing the number of nodes) as H∼αlogN. Moreover, the rate of growth α tends to be similar for all prefectures. Such a fact can clearly be observed in the left and centre plots within Figure 1a,b, where five representative prefectures are highlighted, from largest to smallest in terms of GDP (gross domestic product) size and selected on similar ranking intervals.

In contrast, the mutual information *I* exhibits a more complex, non linear, behaviour as shown in plot (c). One can note that *I* tends to decrease for the very large prefectures (i.e., Tokyo, Fukuoka) as the number of companies, (i.e., nodes) and trades (i.e., edges) increase. However, similar behaviour is not fully replicated in smaller prefectures (i.e., Kagoshima, Wakayama, and Tottori), where *I* may be increasing, stable, or decreasing. Moreover, and distinctly from *H* or *J*, the specific and numerical value of *I* bear a much weaker, albeit yet existing, relation to the system size as will be demonstrated further on, within the subsection allometric nature of the mutual information.

#### 2.1.2. The Geographical Perspective of Mutual Information

As described above, comparison among prefectures of the numerical value of the total mutual information *I* at a given time gives little way of immediate insight, and their relative significances can only be appreciated once the association with metabolism as described by the framework within the next subsection, the allometric nature of the mutual information, is in place. However, a clear picture also emerges once a geospatial perspective is combined with a time series vector analysis for *I*, where the average rate of decline for each prefecture is linearly obtained by fitting $I_{t}=a+b\left(t-t_{0}\right)$.

**Figure 2:**
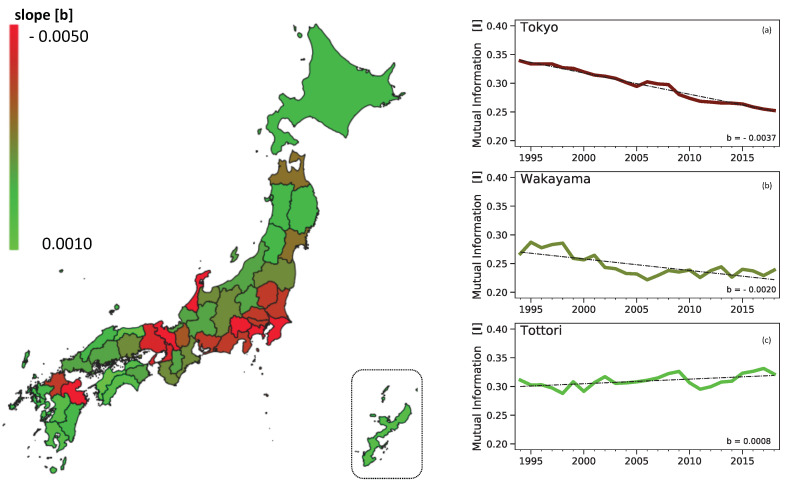
Average decline/increase rate of the mutual information within prefectures in Japan, 1994 to 2018. The map on the left consists of a geographical heatmap for the average yearly rate of decline (**red**) or increase (**light green**) of the mutual information for each of the 47 prefectures as approximated by a linear fitting $I_{t}=a+b\left(t-t_{0}\right)$. Each graph on the side corresponds to the evolution of the total mutual information *I* (y-axis) over the period 1994–2018 (x-axis) for (**a**) Tokyo, (**b**) Wakayama and (**c**) Tottori prefectures.

The heatmap of Japan within Figure 2 shows the geographical distribution of the average rate of decline (b<0) or increase (b>0) to the mutual information *I* over the period in study, 1994–2018. It is easy to visualise that the highest rates of declines (i.e., red areas) are almost totally associated with the prefectures and urban conurbations of Japan’s largest cities, with the sole exceptions of (a) Sapporo, a large city in a very large rural prefecture (Hokkaido) and (b) Oita where no immediate explanation can be found. In a consistent manner, the lowest rates of decline (or slight increase) are associated with the smallest prefectures, in economic terms as measured by the GDP, such as Tottori and Ehime. These results suggest that a time series analysis of the evolution of the mutual information provides a measure (the linear slope ‘*b*’ or the average changes to *I*), which reflects the level of the economic activity, or urbanism, for a given region. Essentially this approach can be feasibly used to potentially define economic clusters and conurbations in a quantitative manner through a single unit measure.

## 3. The Allometric Nature of Mutual Information

**Figure 3:**
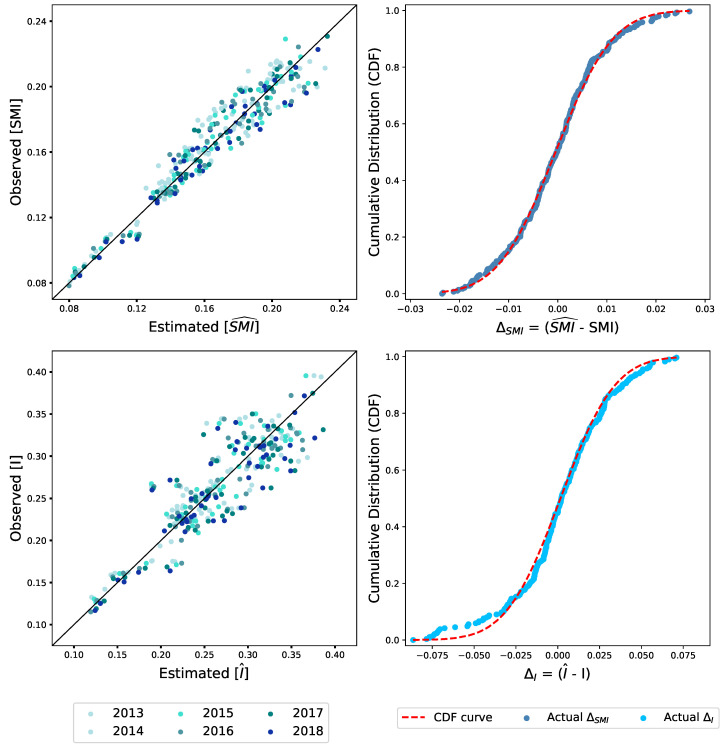
Observed and estimated values for the structural mutual information SMI and total mutual information *I* for the years 2013–2018. The **top left** panel shows the comparative results between the values observed for structural mutual information SMI (or $\tilde{I}$ as described within Methods) on the y-axis against those estimated by making use of Equation (11) on the x-axis. Each dot consists of a single prefecture within Japan at a specific year, with years colour mapped from lighter to darker shades, older to the most recent. The diagonal line represents the point where y=x. Similarly, the **bottom left** panel shows the observed mutual information *I* on the y-axis, calculated in accordance with Equation (7), against those estimated by the model by making use of Equation (12) on the x-axis. On the **right side**, the differences between the estimates to the actual values (x-axes) are ranked and plotted against the cumulative function of a normally distributed curve as shown by the red lines.

The results shown by each of the panels within Figure 3 lend important weight to the validation of a framework to quantify and evaluate mutual information within networks through the prism of biological metabolism and allometric scaling.

Firstly the distinction between structural mutual information SMI (or $\tilde{I}$ as explained within the Methods section) and total mutual information *I* and an analogy to basal metabolism and physical metabolism provide a useful description of the important differences between information (a) arising simply as a result of the existence of a node or company type and (b) that results from the dynamics and interaction between agents.

Within this context, it is to a certain extent remarkable to note the emergence of the 3/4 allometric scaling coefficient, as indicated (see Methods) by the results within Figure 3, and that by applying the same coefficients to both types of mutual information, SMI and *I*, one can observe that the distribution of variance is larger for the ‘total metabolism’ and are relatively small for the ‘resting metabolism’. Moreover, such distributions of variance fit reasonably well to normally distributed curves (albeit with some differences towards the tail values) which indicate that such a variance is consistent with a generic random stochastic process. Here, we note that the number of datapoints are relatively limited, around 280, which can exaggerate the effects towards the tail.

Secondly, the framework and scaling of mutual information provide us with a valuable insight in terms of the economic structure of the prefectures: The ‘structural efficiency’ of a prefecture is not determined by size in isolation (as measured by the entropy) but by the diversity of the agent types within the system (which can be captured by SMI). Here it is important to pause and explain ‘structural efficiency’.

Within any business environment, a company would ideally like to sell products to every other company as it would increase sales and profits. However, resource limitation and costs of trading with various parties lead to a selection of business partners or ‘preference to trade’. Therefore, from a narrow perspective, the more a company sells to a single partner, the lower the acquisition costs. However, one can argue that such an approach also leads to significant inefficiency within an economic system since opportunities for better and innovative trading and new links are reduced. Therefore, SMI and *I* can be effectively viewed as an indicator as to the distance of a prefecture to a theoretical free market configuration.

### Entropy and Mutual Information Micro Features

Whereas analysing the evolution of the mutual information from a macro level provides an insight with regards to the structural economic activity of prefectures, by zooming into the local, micro level, structures of interactions it is possible to better understand the impact of the essential mechanisms underpinning the network in study.

**Figure 4:**
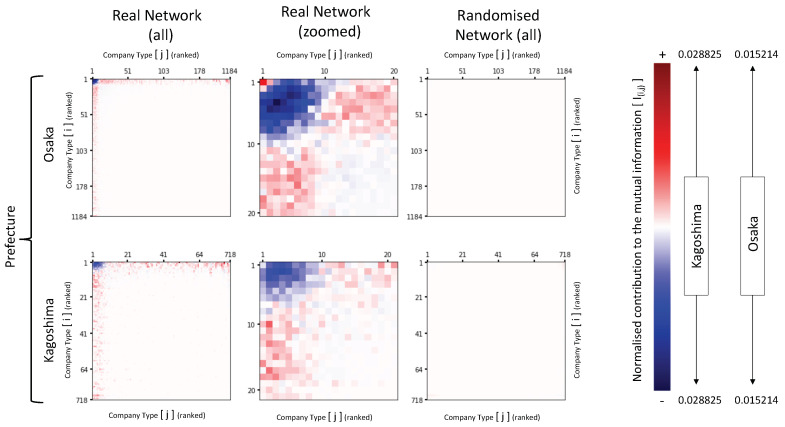
Pointwise contribution to the mutual information, $I_{\left(i,j\right)}$, for real and randomised networks. Each panel represents a heatmap of the pointwise contribution to mutual information for the directional edge combination ‘*i*’ (vertical axis) to ‘*j*’ (horizontal axis), calculated in accordance with Equation (8). Both axes are equal in value, consisting of the ranked sequence of the total degree distribution of companies for the relevant representative prefectures, Osaka in the top row and Kagoshima in the bottom row. The **left** (all degrees) and **centre** (zoomed degrees up to 20) panels show the contribution to the mutual information for the real network, whereas the **right** panels show contribution related to the randomised network. The colour maps on the right show the intensity of the contribution, with different scales by prefecture, but the same for all panels for the selected prefecture. Darker colours are associated with higher numbers with blue being negative values, red being positive, and totally white being zero.

The effect of the preferential attachment and cumulative advantage mechanisms as catalysers to the generation of mutual information can be clearly observed by analysing Figure 4. The pointwise contribution to the mutual information heatmaps for the real networks (left side), within Osaka and Kagoshima prefectures, show the larger absolute values to be concentrated at the left and top borders of the panels. In contrast, the equivalent maps (maintaining the same colour coding scales) for the randomised network show a much more homogenous distribution of values across the heatmaps, with contributions to the mutual information and pointwise values tending towards zero. Moreover, the zoomed maps for the real network (central panels) provide a neat illustration of the core relationship between the cumulative advantage and preferential attachment mechanisms and mutual information: By ‘preferring’ to attach to larger companies, smaller entities tend to ‘repel’ its own kind. As a result, the pointwise mutual information turns negative within the small to small region. In a consistent manner, higher levels of pointwise values tend to be stronger at the preference region (i.e., smaller to larger companies).

**Figure 5:**
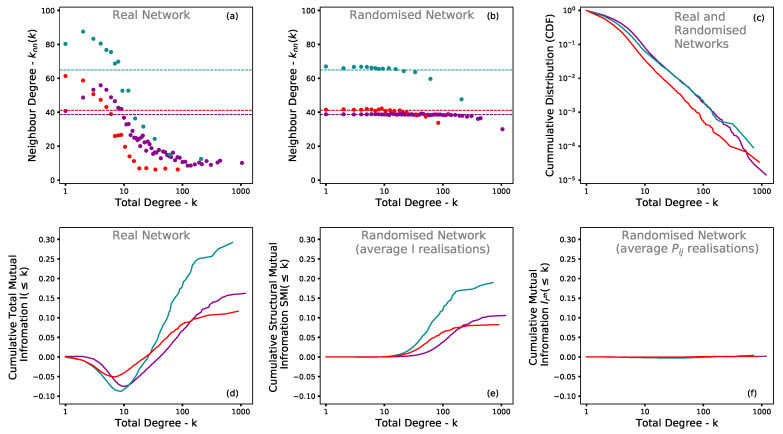
Average degree, population distribution, and cumulative mutual information values for selected Japanese prefectures in 2018. Panels (**a**,**b**) show the average degree of the neighbouring nodes [23] ‘$k_{nn}\left(k\right)$’ (y-axis) of companies with total degree ‘*k*’ (x-axis, on a lognormal scale) for three selected prefectures: Osaka (magenta), Kagoshima (turquoise), and Saitama (red). Each dot represents the aggregate of companies of total degree ’*k*’ and the average of their neighbours ‘$k_{nn}\left(k\right)$’ generated through a binning process with a minimum of 1000 edges (i.e., datapoints) per bin. Whereas (**a**) relates to data extracted directly from the real network, (**b**) shows the average values for 1000 randomised realisations. Panel (**c**) consists of the total degree distribution of companies for the selected prefectures plotted on a log-log scale. The bottom panels (**d,e** and **f**) show the cumulative of the total mutual information *I* within the y-axis as a function of the degree distributions of companies, within the x-axis, on a lognormal scale. The left panel (**d**) relates to data from the real network, whereas the centre panels (**e**) consists of the average value of the mutual information *I* for each of the 1000 realisations adopting $P_{ij}=w_{ij}$ as calculated by Equations (3) and (7). In contrast, the right panel (**f**) consists of the calculation of a single value for mutual information $I_{r^{th}}$ for all aggregated realisations of $P_{ij}$ as described in Equation (10).

However, it is important to note at this stage that by preserving the degree distribution of the nodes and at the same time maintaining the same number of edges, the randomisation process significantly reduces, but does not fully eliminate, the disassortative structure of the network, as shown in (a.2) and (c.2) within Figure 5. This is due to the fact that the neutral degree correlation under a power law degree distribution can only be achieved if the condition described in Equation (9) is satisfied. Therefore, the remaining level of disassortativeness can be regarded as ‘structural’, being the consequence of the power law distribution, since it is simply a fact that such a distribution of companies and company types do not allow for the probabilities of selection of all source and target nodes to be equal.

## 4. Conclusions and Discussion

The results of this research indicated that the allometric scaling of the mutual information within the Japanese interfirm business networks to be akin and analogous to the metabolic rates of biological systems, providing further substance to the metaphor proposed by West [24] when researching the scaling of phenomena of cities and economies. Moreover, the 3/4 scaling exponent found in biological systems [6], as well some of the dynamics within cities and economies [7], fitted very well within our complexity framework when applied to the Japanese economy at a national as well as regional prefecture level.

By measuring the mutual information at a national and regional prefecture, levels under our framework and method, and evaluating over an extensive time series, it is possible to appreciate the relationship between mutual information and the level of economic activity and urbanism of these prefecture, and therefore to place them into a comparative scale.

Moreover, we identified the structural mutual information SMI as the contribution arising as a result of the structure of the nodes, and segregated from the total mutual information *I*, which also includes the dynamics of interaction between agents. In doing so, we were able to clearly articulate that these quantities essentially represent the distance of a given economic structure from a theoretical free market configuration.

Such a finding helps to articulate a paradox which is essentially a core to today’s economic analysis [25]. Whereas markets are most efficient when all agents are equally informed and have equal competitive chances (essentially there is no existence of preferential attachment and cumulative advantage mechanisms), these dynamics embedded within a capitalist system lead to a monopolistic configuration. Therefore, one could reason that in order to promote and protect free markets, governments and related agencies must actually intervene to mitigate the impact of the above mechanisms, and therefore be compelled to negate the more extreme interpretations of ‘invisible hand’ and ‘laissez faire economics’.

From a micro level perspective, the analysis of the pointwise contribution to the mutual information showed that small companies tended to ‘repeal’ each other and be dependent on large entities. Again, one could argue that such a configuration is contrary to the efficient, free market configuration, and therefore has a potential focus to enhanced economic policy.

## 5. Materials and Methods

### 5.1. Measuring Entropy and Mutual Information

Individual companies *c* within the set of companies C are aggregated into groups $S_{k}$ of companies with same total degree *k*:

(2) $$S_{k}=\left\{c|k_{c}=k,c\in C\right\}$$

where $k_{c}$ is the total degree of company *c*.

By making an analogy to the real biological ecosystems, one may regard these groups as representing the average body size or mass of the individuals within that given group, since the total degree of companies scale in accordance with their sizes [11,13] within the business context, as measured by the number of employees, income, or total assets.

The expectation of edges between two groups $S_{i}$ and $S_{k}$, or body sizes, is:

(3) $$w_{ij}=\frac{E_{ij}}{E}$$

where $E_{ij}$ is the total number of edges within the network from the source group *i* to the target group to *j*. The sum of these edges represents a proxy for the direct flow of resources between different groups. *E* is the total number of edges within the network. Within this configuration, the expectation *w* is taken as $P_{ij}\approx w_{ij}$. Therefore, in a similar manner, the probability of encountering an edge starting from a node of degree *i* within the distribution of the total population of *E* edges is:

(4) $$P_{i}^{\left(\mathrm{out}\right)}\approx W_{i}=\frac{\sum_{x=1}^{k_{max}}E_{ix}}{E}\;,\;P_{i}^{\left(\mathrm{in}\right)}\approx W_{i}=\frac{\sum_{x=1}^{k_{max}}E_{xi}}{E}.$$

The source $H^{\left(\mathrm{out}\right)}$, target $H^{\left(\mathrm{in}\right)}$, and total *H* entropies for both real and randomised networks in Japan and each of its 47 prefectures in isolation, are calculated in accordance with the classic Shannon construct:

(5) $$H^{\left(\mathrm{out}\right)}=-\sum_{i}P_{i}^{\left(\mathrm{out}\right)}log_{2}P_{i}^{\left(\mathrm{out}\right)}\;,\;H^{\left(\mathrm{in}\right)}=-\sum_{i}P_{j}^{\left(\mathrm{in}\right)}log_{2}P_{j}^{\left(\mathrm{in}\right)}\;,\;H=H^{\left(\mathrm{out}\right)}+H^{\left(\mathrm{in}\right)}\;.$$

The logarithm base of two intends to represent the discrete and binary nature of undirected and unweighted edges, i.e., either two selected companies transact to each other or not.

In a similar manner, the joint entropy for two groups *i* trading with *j*, is given by:

(6) $$J=-\sum_{i,j}P_{ij}log_{2}P_{ij}$$

with the corresponding mutual information is given by:

(7) $$I=-\sum_{i,j}P_{ij}log_{2}\frac{P_{ij}}{P_{i}^{\left(\mathrm{out}\right)}P_{j}^{\left(\mathrm{in}\right)}}$$

and each pointwise contribution to the mutual information being:

(8) $$I_{\left(i,j\right)}=-P_{ij}log_{2}\frac{P_{ij}}{P_{i}^{\left(\mathrm{out}\right)}P_{j}^{\left(\mathrm{in}\right)}}.$$

### 5.2. Network Randomisation and Rewiring Process

Here, we make use of the symbol ($\tilde{}$) to denote quantities and corresponding outputs from Equations (2)–(8) associated with the randomised and rewired network. The process consists in generating a directional edge between two companies, namely the source and target nodes, for each step until the total number of edges $\tilde{E}=E$ is achieved. A constraint is placed whereby the total degree of each node within the population is maintained so that $\tilde{k_{c}}=k_{c}$ and $\tilde{S_{k}}=S_{k}$. In this manner, the degree distribution is preserved but the degree correlation of nodes is effectively and mostly randomised. We note, however, that the process as a whole is not totally random (i.e., without any form of correlations) since the node population constraint leads to slight distortions on the probability of edge selections, since the condition:

(9) $$NP_{\alpha }\alpha =NP_{\beta }\beta =...=NP_{\omega }\omega ,\;\mathrm{where}\left\{\begin{array}{c} N\;\mathrm{is}\;\mathrm{the}\;\mathrm{total}\;\mathrm{number}\;\mathrm{of}\;\mathrm{nodes}\\ \left\{\alpha ,\beta ,...,\omega \right\}\;\mathrm{is}\;\mathrm{the}\;\mathrm{set}\;\mathrm{of}\;\mathrm{the}\;\mathrm{total}\;\mathrm{degree}\;\mathrm{of}\;\mathrm{the}\;\mathrm{nodes}\\ \;\;\mathrm{within}\;\mathrm{the}\;\mathrm{population}\\ \left\{P_{\alpha },P_{\beta },...,P_{\omega }\right\},\;\mathrm{the}\;\mathrm{related}\;\mathrm{set}\;\mathrm{of}\;\mathrm{the}\;\mathrm{probability}\;\mathrm{of}\\ \;\;\mathrm{nodes}\;\mathrm{for}\;a\;\mathrm{given}\;\mathrm{total}\;\mathrm{degree} \end{array}\right.$$

is not satisfied for every and any power law distribution represented by $P_{k}∼k^{-\gamma }$, where γ≠1.

Therefore, we define the observed structural mutual information SMI as being the mutual information for the rewired network $\tilde{I}$ (i.e., SMI = $\tilde{I}$) computed in accordance with Equations (3)–(8) above with the equivalent quantities of the rewired network.

Furthermore, we also estimate the probabilities from the outcomes of ρ=1000 realisations in the following way:

(10) $$P_{ij}=\frac{\sum_{r=1}^{R}E_{ij}^{r}}{E*\rho }\;,\;\mathrm{where}\;E_{ij}^{r}\;\mathrm{is}\;\mathrm{the}\;\mathrm{value}\;\mathrm{of}\;E_{ij}\;\mathrm{in}\;\mathrm{the}\;\rho^{th}\;\mathrm{realisation}.$$

As shown in (c.3) within Figure 5, the degree correlation and related mutual information $I_{r^{th}}$ in this circumstance tends to zero as expected. The comparison between finite statistic and continuum realisation methods illustrates the effect of applying the analysis of a single realisation only to a real world network.

### 5.3. Methods Underpinning the Complexity Framework

As previously described within the Introduction section, the framework draws parallels between the allometric scaling of metabolic rates and the mutual information obtained from the method applied to the network. This is done in context of a dataset underpinning a real financial and economic system.

#### 5.3.1. Structural Mutual Information, SMI

As described within the Introduction, the structural mutual information SMI is intended to capture the basic quantities held by the network simply as a result of the power law like degree distribution of companies, and their related sizes, through a method inspired by the allometric scaling in ecological systems.

Therefore, our method adapts the allometric scaling Equation (1) to the context of our research, specifically a trade network between companies in different territories. We regard the average degree of each network, namely the total number of trade links *E* (i.e., the total money flows) divided by the total number of companies |C| to be equivalent to the supply rate, and therefore R∝E/|C|. In a similar manner, the variability affecting body size can be represented respectively by the ratio between the diversity of species (i.e., total number of groups) and the number of links within the network, and therefore $Q_{1}\propto S/E$. Lastly, we take the equivalent of the body size quantity of a prefecture to be proportional to the largest total degree (i.e., the sum of a node’s in and out degrees) in which in turn it is proportional to the number of groups, and therefore $M\propto k_{max}\propto S$. By substituting these elements into Equation (1), using a single proportional constant λ, and adding a minimum floor parameter, we obtain:

(11) $$\hat{SMI}=\frac{\lambda }{\left|C\right|}S^{7/4}+\tau$$

where λ∼0.6 and τ∼0.0275 are empirically derived from the data, and b∼3/4 is also corroborated by the data.

#### 5.3.2. Total Mutual Information I

As described within the Introduction, the total mutual information *I* can be regarded a multiplier to the core, structural mutual information SMI. Therefore, it can be mathematically represented as:

(12) $$\hat{I}=\kappa \hat{SMI}+\left(1-\kappa \right)\tau =\frac{\kappa \lambda }{\left|C\right|}S^{7/4}+\tau$$

where it is empirically found that κ∼7/4.

## Abbreviations

The following abbreviations are used in this manuscript:

SMI	Structural Mutual Information
I	Total Mutual Information
H	Entropy
J	Total Joint Entropy
GDP	Gross Domestic Product
